# Supplementary material for: Symport and antiport mechanisms of human glutamate transporters
Source: Nat Commun. 2023 May 4;14:2579. doi: 10.1038/s41467-023-38120-5 (PMC10160106; doi:10.1038/s41467-023-38120-5)
Supplement: Supplementary file 1 — Supplementary Information [file 41467_2023_38120_MOESM1_ESM.docx]

**Symport and antiport mechanisms of human glutamate transporters**

Biao Qiu^1,*^, Olga Boudker^1,2,*^

^1^ Department of Physiology & Biophysics, Weill Cornell Medicine, 1300 York Ave, New York, NY 10021, USA

^2^ Howard Hughes Medical Institute, Weill Cornell Medicine, 1300 York Ave, New York, NY 10021, USA

^*^ Correspondence: [biq2001@med.cornell.edu](mailto:biq2001@med.cornell.edu), olb2003@med.cornell.edu

**This PDF file includes the following:**

Supplementary Figures 1-8

Supplementary Tables 1-4

**
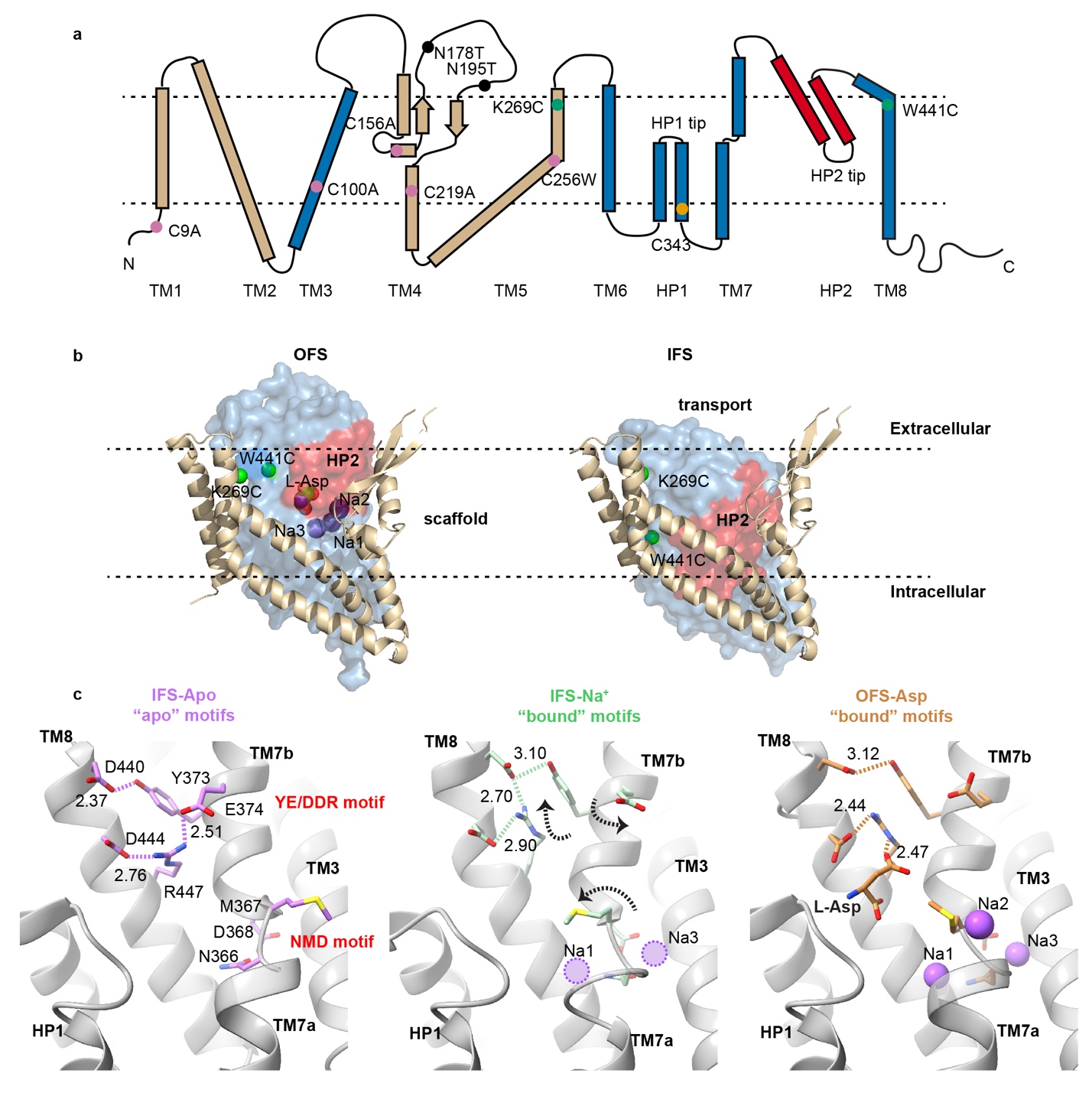
**

**Supplementary Figure 1: EAAT3 topology, elevator transport mechanism, and "dancing” motifs.** (**a**) The EAAT3 topology with scaffold domain colored wheat, transport domain blue, and HP2 red. The pink circles represent cysteines mutated to alanines or a tryptophan; the orange circle corresponds to the highly conserved cysteine in HP1; the green circles represent K269 and W441 mutated to cysteine for crosslinking; the loops connecting two α-helical segments in HP1 and HP2 are labeled “HP1 tip” and “HP2 tip”. (**b**) The elevator movements of the transport domain. Shown are single hEAAT3g protomers in the outward-facing aspartate-bound state (OFS, left, PDB ID: 6X2Z [http://doi.org/10.2210/pdb6x2z/pdb]) and inward-facing sodium-bound state (IFS, right, PDB ID: 6X2L [http://doi.org/10.2210/pdb6x2l/pdb]). The scaffold and transport domains are in a cartoon and surface representation, respectively. Green spheres emphasize K269 and W441 Cα-s. The bound aspartate and sodium ions are shown as spheres and colored by atom type. (**c**) The “dancing” NMD and YE/DDR motifs form the substrate- and ion-binding sites in IFS-Apo (left, PDB ID: 6X3F [http://doi.org/10.2210/pdb6x3f/pdb]), IFS-Na^+^ (middle, PDB ID: 6X2L), and OFS-Asp (right, PDB ID: 6X2Z) states. HP2, occluding the sites, is removed for clarity. Dashed lines mark key interactions (distance in Å) in the motifs, which transition from an “apo” to a “bound” configuration upon sodium binding, prepping the sites for the consequent transmitter binding. The dotted circles represent bound unresolved Na^+^ ions in IFS-Na^+^.

**
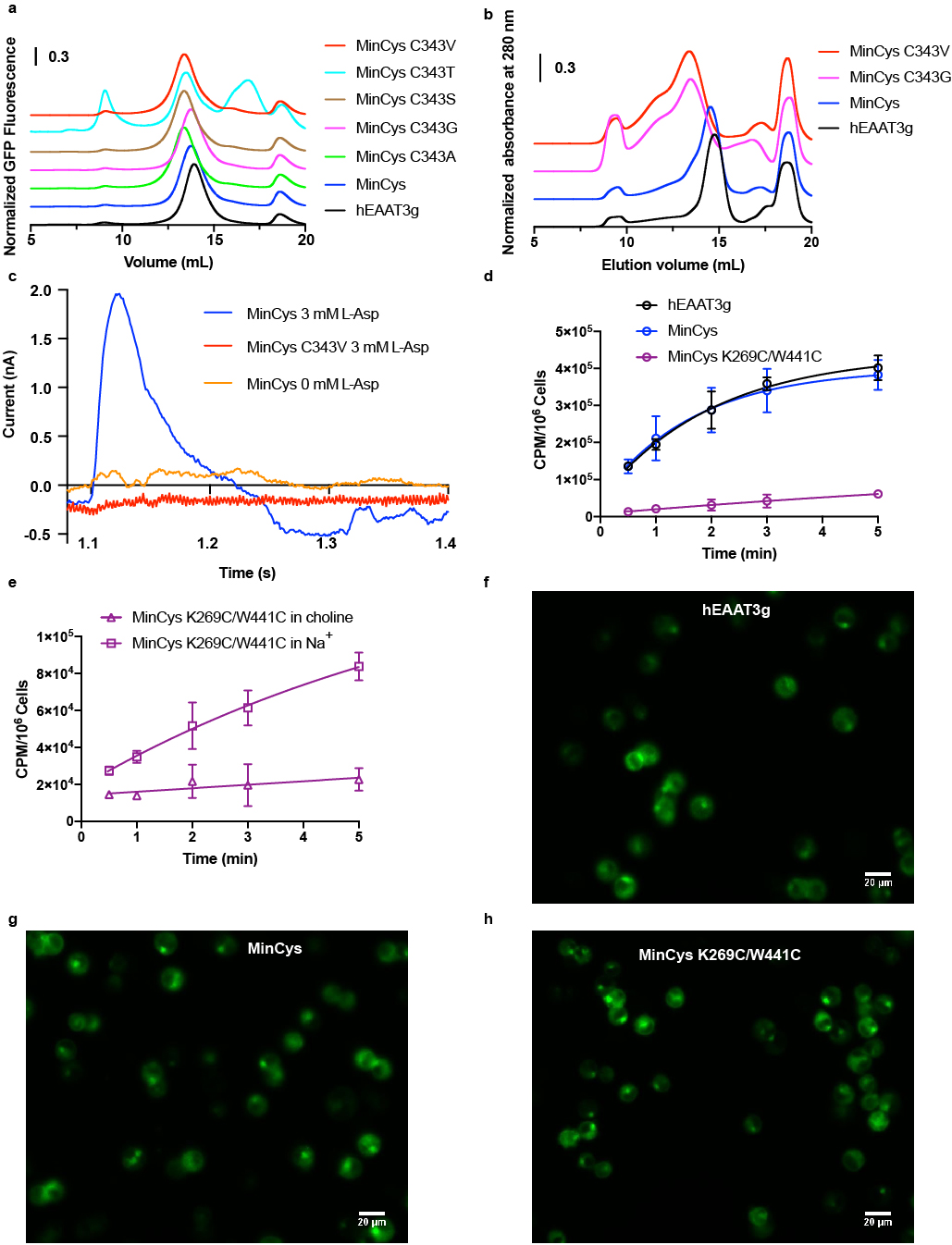
**

**Supplementary Figure 2: Characterization of MinCys and MinCys K269C/W441C EAAT3.** (**a**), Normalized fluorescence size exclusion chromatography (FSEC) profiles of hEAAT3g (black), MinCys (blue), and MinCys with the following additional mutations: C343A (green), C343G (magenta), C343S (brown), C343T (cyan), and C343V (red). Only MinCys and its C343G and C343V mutants show elution profiles similar to hEAAT3g. Other C343 mutants show unfavorable behaviors indicative of denatured proteins. (**b**), Preparative SEC elution profiles of hEAAT3g (black), MinCys (blue), MinCys C343V (red), and C343G (magenta). The peaks eluting at ~17 and 19 ml are the PreScission protease and the cleaved Strep II-GFP tag, respectively. The absorbance at 280 nm was normalized by the peak maximum. The SEC profiles of MinCys C343V and C343G indicate that proteins form heterogeneous oligomers and aggregates. (**c**) MinCys displays substrate transport currents (blue) in solid-supported membrane (SSM) assays. In contrast, the C343V mutant (red) is indistinguishable from the control (orange). (**d**), Radio-labeled [3H]L-aspartate uptake into HEK293 cells expressing hEAAT3g (black), MinCys (blue), and MinCys K269C/W441C (purple). The background determined without the sodium gradient was subtracted from the data. (**e**), The substrate uptake of MinCys K269C/W441C in NaCl (squares) and choline chloride (triangles). The lines through the data in panels (e) and (d) are to guide the eye. The substrate uptake assays were performed in triplicate, with two technical repeats each. The error bars show the standard deviations of three independent measurements. (**f, e, h**), Fluorescence imaging of the cells used in the uptake experiments showed similar protein expression and localization of the protein constructs. Representative images are shown. Source data are provided as a source data file.

**
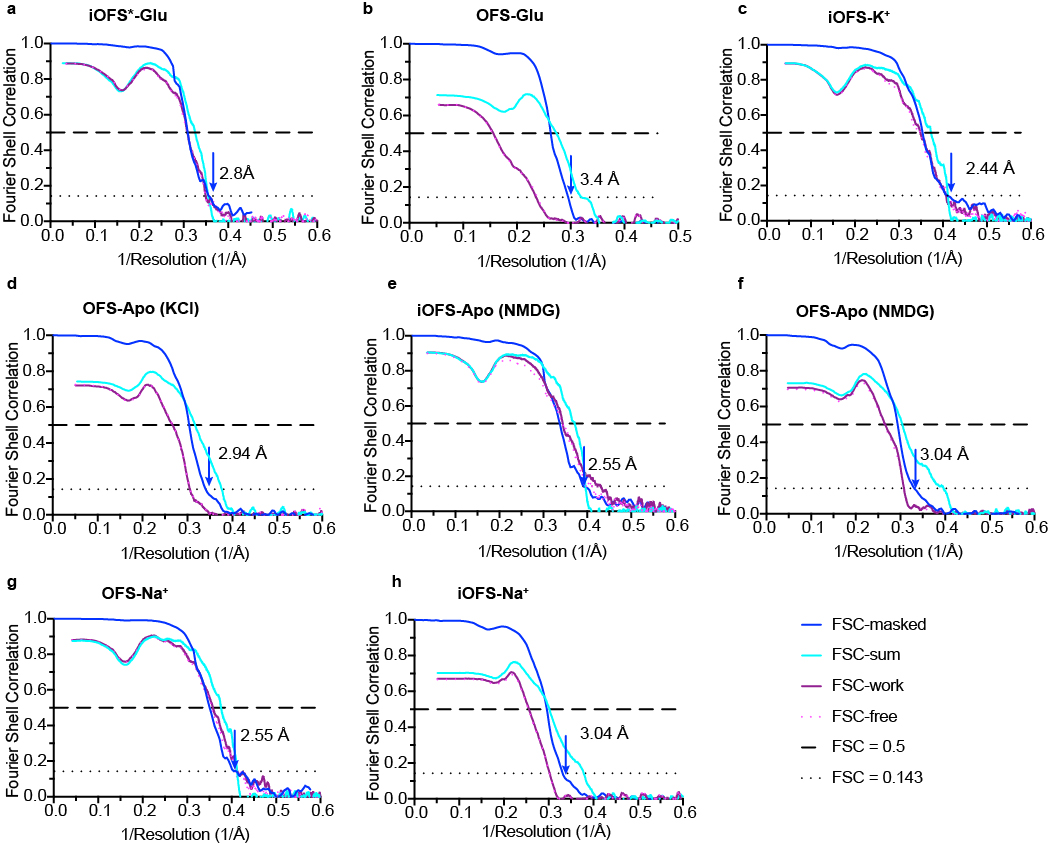
**

**Supplementary Figure 3: Map and model validations**. Fourier shell correlation (FSC) curves for the density maps and map and model validations for EAAT3-X in the following states: iOFS^*^-Glu (**a**), OFS-Glu (**b**), iOFS-K^+^ (**c**), OFS-Apo in KCl (**d**), iOFS-Apo in NMDG (**e**), OFS-Apo in NMDG (**f**), OFS-Na^+^ (**g**), and iOFS-Na^+^ (**h**). Shown are the FSC curves for the density maps (blue), the FSC curves for the refined models versus full maps (cyan), and half maps for cross-validation (purple and pink dots). Dashed and dotted lines correspond to FSC values of 0.5 and 0.143, respectively.

**
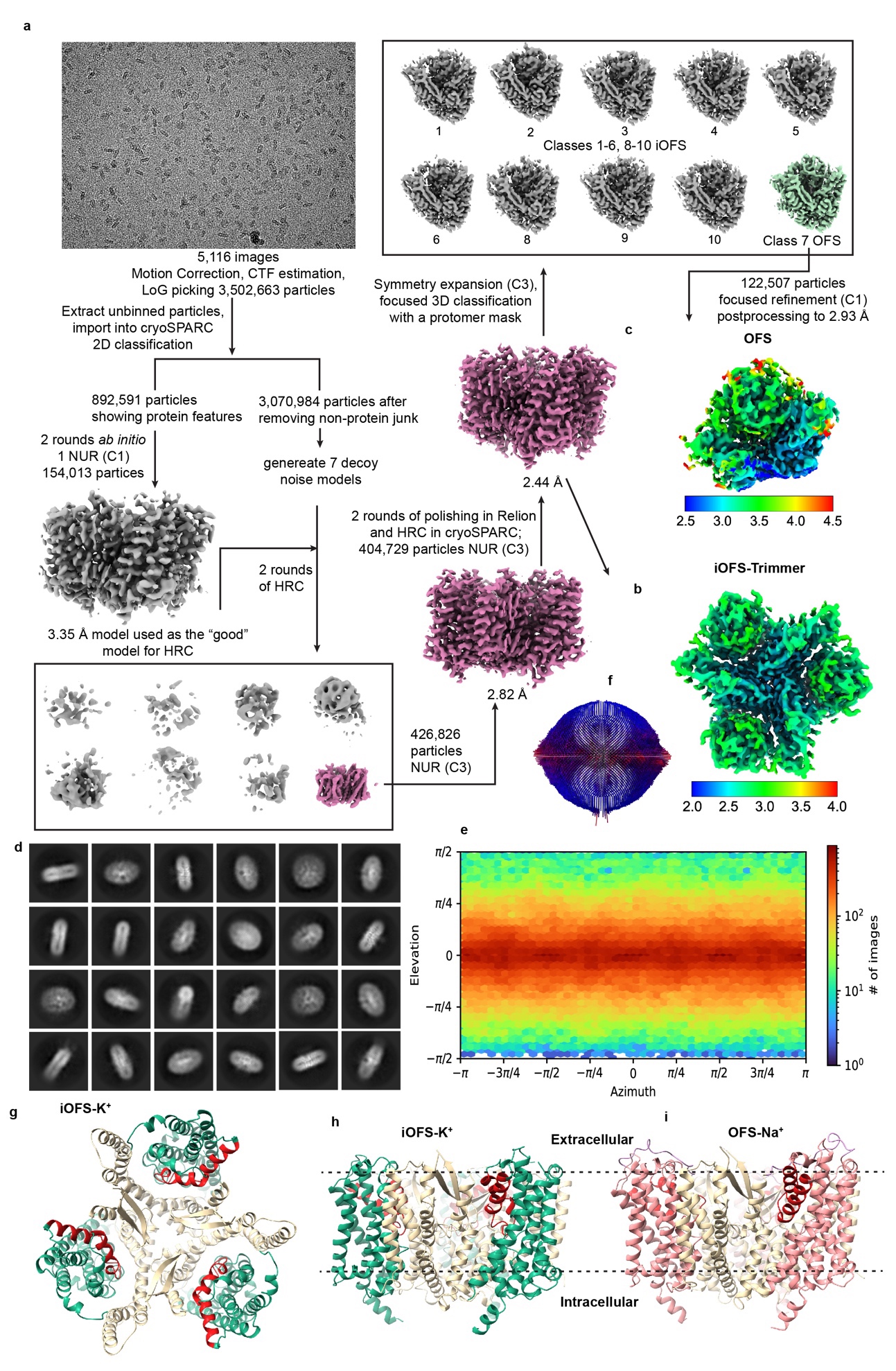
**

**Supplementary Figure 4: A data processing flowchart for EAAT3-X in 300 mM KCl and iOFS and OFS models of EAAT3-X.** (**a**), An example of an aligned image and the data processing flowchart. The iOFS-K^+^ map refined in C3 (**b**) and the OFS-Apo protomer map (**c**) are colored by the local resolution. (**d**), The representative 2D averages. (**e**, **f**), The angular distributions of particles for the 3D reconstitutions of the iOFS trimer using cryoSPARC v3.2.0 (**e**) and the OFS protomer using Relion 3.1.0. The top (**g**) and side (**h**) views of the iOFS-K^+^ trimer, with the scaffold domain in wheat, the transport domain in green, and HP2 in red. (**i**), The side view of the iOFS-Na^+^ trimer, with the scaffold domain in wheat, the transport domain in pink, and HP2 in red. Panels (**h**) and (**i**) illustrate the different positions of the transport domain in the iOFS and OFS.

**
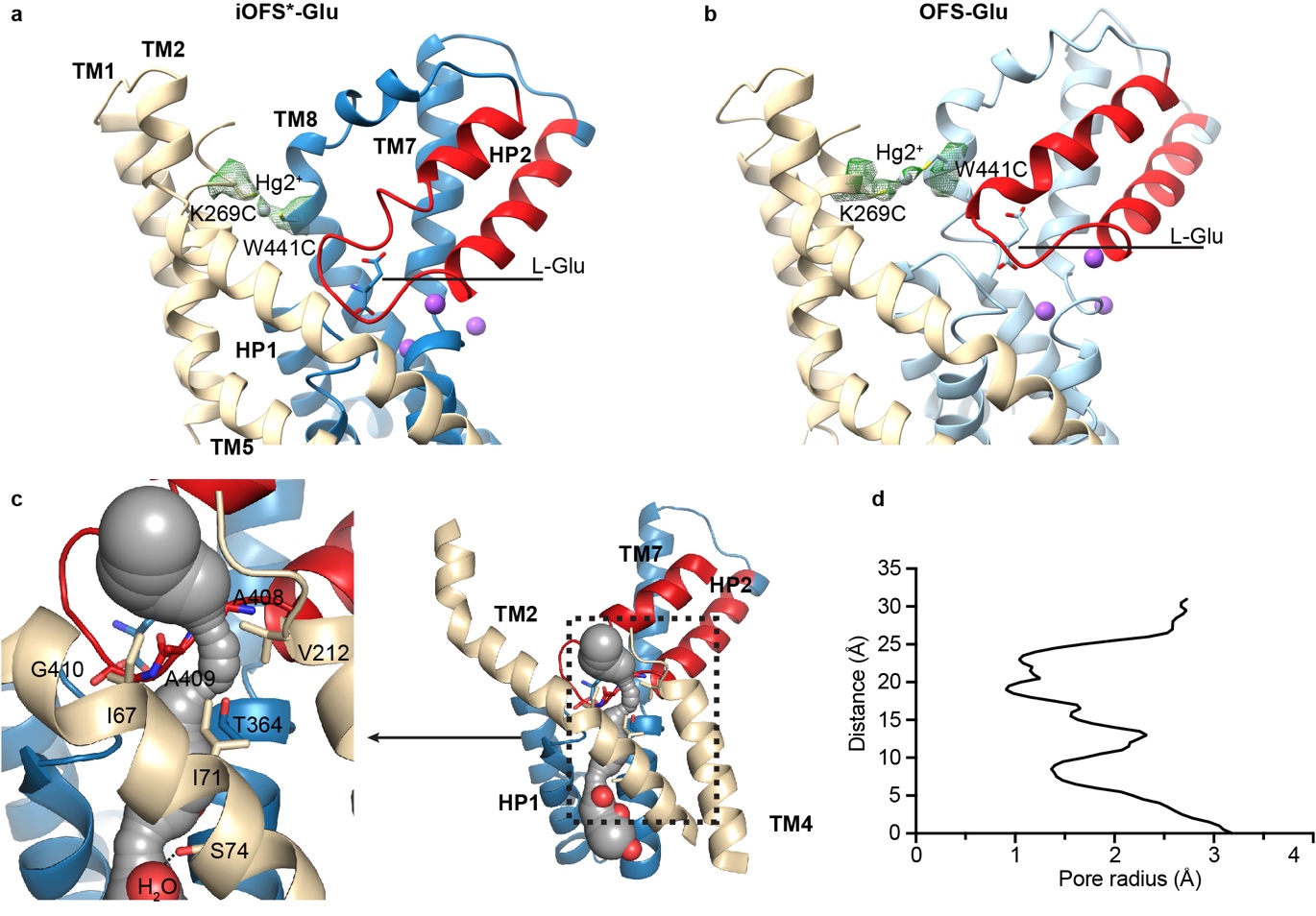
**

**Supplementary Figure 5: Glutamate-bound EAAT3-X in iOFS^*^ and OFS.** A cross-link between K269C and W441C residues in iOFS^*^-Glu (**a**) and OFS-Glu (**b**). Single protomers are shown in cartoon representation colored as in Supplementary Figure 1, with TMs 3, 4, and 6 removed for clarity. The green mesh is the EM density around K269C, W441C, and Hg^2+^ ions contoured at 5 σ. (**c**), The tunnel in iOFS^*^-Glu is calculated by CAVER 3.0 and shown as gray spheres. The observed solvent molecules (red spheres) are visible in the lower part of the tunnel. Residues forming the extracellular constriction and S74 implicated in anion selectivity are shown as sticks. (**d)**, The pore radius along the tunnel. Zero on the Y-axis is set to the location of the second water in the cytoplasmic vestibule.

**
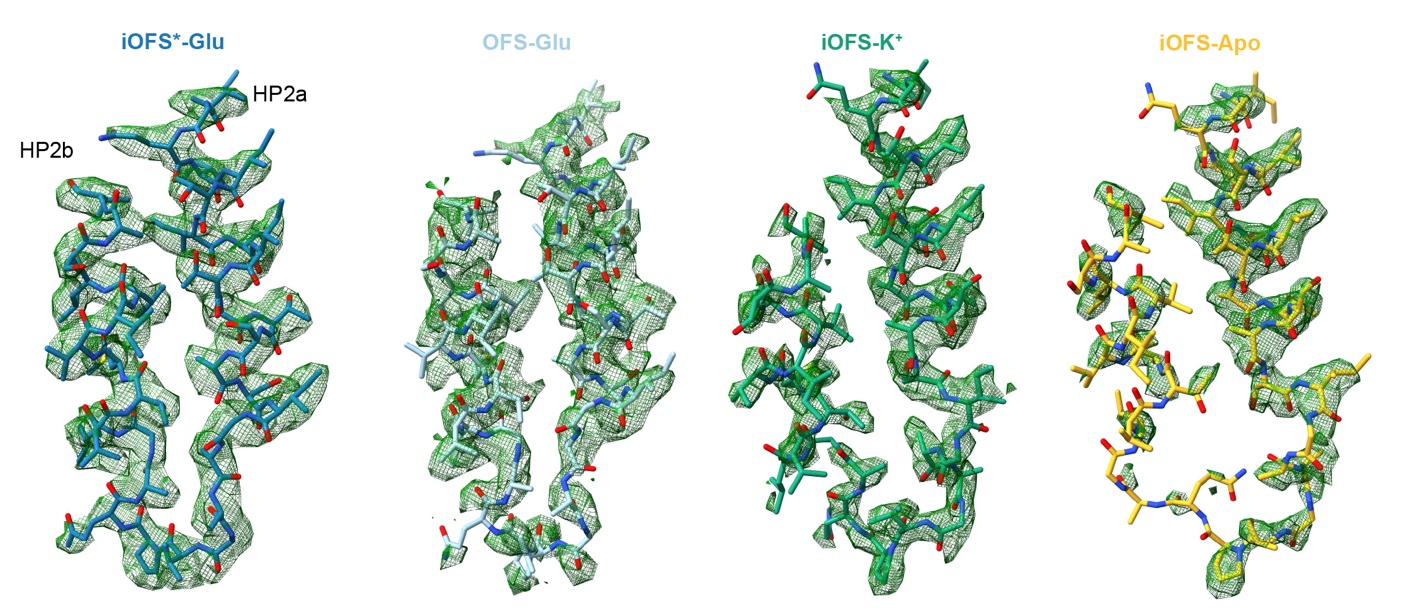
**

**Supplementary Figure 6: HP2 EM density maps.** The maps, shown as green mesh, are contoured at 5 σ around HP2 of, from left to right, iOFS^*^-Glu, OFS-Glu, iOFS-K^+^, and iOFS-Apo states. Modeled HP2 is shown in stick representation.

**
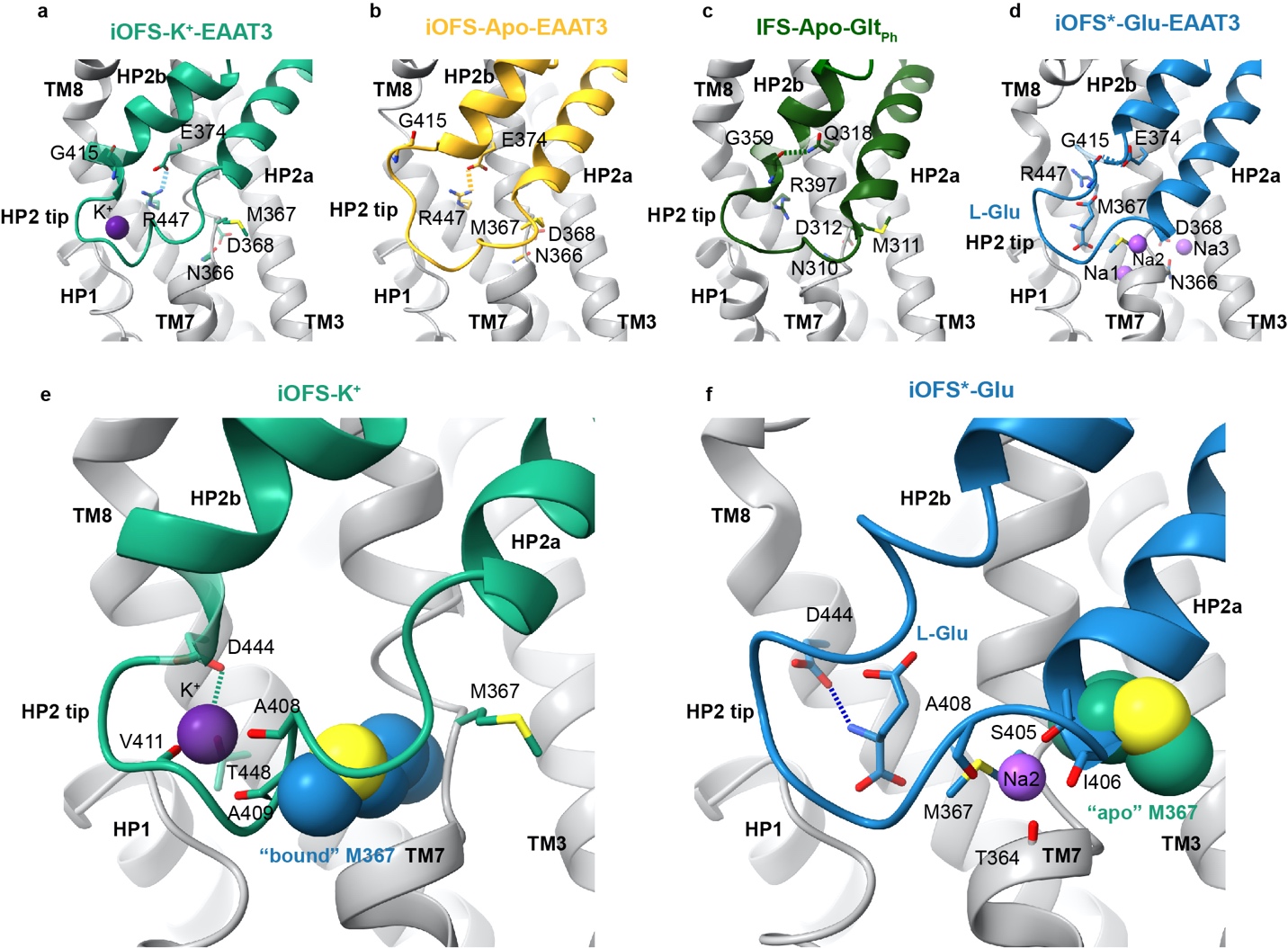
**

**Supplementary Figure 7: The NMD and YE/DDR motifs mediate ion coupling in EAAT3.** (**a-d**), The YE/DDR motifs in EAAT3 iOFS-K^+^, EAAT3 iOFS-Apo, Glt_Ph_-Apo, and EAAT3 iOFS^*^-Glu. Structures are superposed on the cytoplasmic halves of their transport domains (residues 314-372 and 442-465 for EAAT and residues 259-317 and 392-415 for Glt_Ph_, PDB ID: 4P1A [http://doi.org/10.2210/pdb4p1a/pdb]). The interactions between E374 and R447 or G415 in EAAT3 and between corresponding Q318 and G359 in Glt_Ph_ are shown as dashed lines. (**e**, **f**), The orientation of M367 in the NMD motif determines whether HP2 can coordinate the K^+^ ion or L-Glu/Na2. (**e**), HP2 in the potassium-bound conformation (green) would sterically clash with M367 in the glutamate-bound conformation (dark blue spheres labeled “bound” M376). M367 in this conformation (shown as sticks) points away from HP2. (**f**), HP2 in the glutamate-bound conformation would clash with M367 in the apo conformation (green spheres labeled “apo” M367). In this state, M367 (shown as sticks) is sandwiched between L-Glu and Na2, with its sulfur atom coordinating the ion. Transport domains of iOFS-K^+^ and iOFS^*^-Glu were superimposed to generate these figures.

**
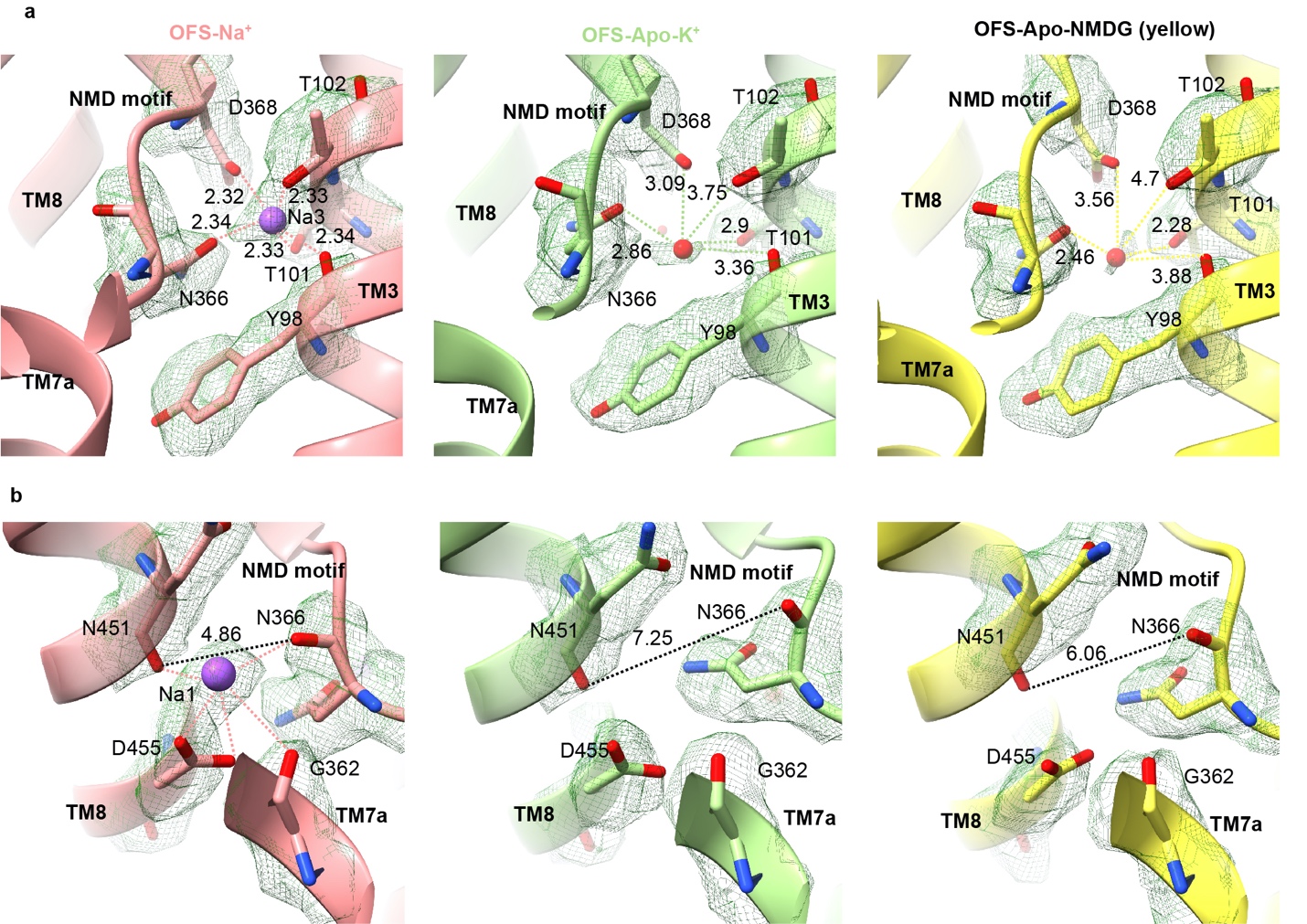
**

**Supplementary Figure 8: Distorted sodium sites in OFS-Apo.** EM density and geometry of Na3 (**a**) and Na1 (**b**) sites of OFS-Na^+^ (left), OFS-Apo_K_ in 300 mM KCl (middle), and OFS-Apo_NMDG_ 150 mM NMDG chloride (right). The density maps, shown as green mesh, are contoured at 5 σ. The dashed yellow lines in OFS-Na^+^ emphasize interactions between the Na^+^ ions and coordinating oxygens. The distances between the Na^+^ ion or water in the Na3 site and the coordinating oxygens are shown in (**a**). In OFS-Apo structures, a solvent molecule replaces the Na^+^ ion in the Na3 site, and the distances to coordinating moieties increase (**a**). The dashed black lines in (**b**) mark the distances between the carbonyl oxygens of N366 and N451. The distance increases between these atoms observed in KCl and NMDG chloride reflect the distortions of the Na1 site in OFS-Apo.

**Supplementary Table 1: Cryo-EM data collection**

|  | EAAT3-X | | | |
| --- | --- | --- | --- | --- |
|  | 20 mM L-Glu | 300 mM KCl | 150 mM NMDG-Cl | 300 mM NaCl |
| Microscope/camera | Krios/K3 | Krios/K3 | Krios/K3 | Krios/K3 |
| Voltage (kV) | 300 | 300 | 300 | 300 |
| Energy filter | 20 eV | 20 eV | 20 eV | 20 eV |
| Magnification | 81,000 X | 105,000 X | 105,000 X | 105,000 X |
| Superresolution pixel size (Å) | 0.5413 | 0.426 | 0.426 | 0.426 |
| Dose (e-/Å2) | 50.27 | 51.10 | 50.73 | 57.52 |
| Number of frames | 40 | 48 | 52 | 40 |
| Exposure time (s) | 2 | 2.4 | 2.6 | 2.4 |
| Defocus range (μm) | -0.8 ~ -2.5 | -1.3 ~ -2.0 | -1.3 ~ -1.6 | -1.0 ~ -1.8 |

**Supplementary Table 2: Cryo-EM data refinement and validation for the major conformation in C3**

|  | iOFS^*^-Glu  EMD-26985  PDB-8CTC | iOFS-K^+^  EMD-26997  PDB-8CUA | iOFS-Apo  EMD-27000  PDB-8CUI | OFS-Na^+^  EMD-27006  PDB-8CV2 |
| --- | --- | --- | --- | --- |
| **Data collection and processing** |  |  |  |  |
| Symmetry imposed | C3 | C3 | C3 | C3 |
| Initial particle images (no.) | 8,511,485 | 3,502,663 | 2,553,613 | 2,949,270 |
| Final particle images (no.) | 496,972 | 404,729 | 210,303 | 519,857 |
| Map resolution (Å)  FSC threshold | 2.80  0.143 | 2.44  0.143 | 2.55  0.143 | 2.44  0.143 |
| Map resolution range (Å) | 2.46 ~ 39.28 | 1.86 ~ 39.27 | 2.23 ~ 39.19 | 2.18 ~ 37.14 |
|  |  |  |  |  |
| **Refinement** |  |  |  |  |
| Initial model used (PDB code) | 6X2Z, 6X2L | 6X2Z, 6X3F | 6X2Z, 6X3F | 6X2Z, 6X2L |
| Model resolution (Å)  FSC threshold | 3.07  0.5 | 2.68  0.5 | 2.69  0.5 | 2.66  0.5 |
| Model resolution range (Å) | 3.07 ~ 36.35 | 2.68 ~ 23.80 | 2.69 ~ 28.84 | 2.66 ~ 23.86 |
| Map sharpening *B* factor (Å^2^) | -138.5 | -93.7 | -92.6 | -98.4 |
| Model composition  Non-hydrogen atoms  Protein residues  Ligands | 9,309  1,218  15 | 9,534  1,248  6 | 9,357  1,230 | 9,882  1,299  6 |
| *B* factors (Å^2^)  Protein  Ligand | 60.47  80.93 | 47.07  101.74 | 52.75 | 51.98  43.28 |
| R.m.s. deviations  Bond lengths (Å)  Bond angles (°) | 0.005  0.736 | 0.005  0.968 | 0.003  0.534 | 0.003  0.533 |
| Validation  MolProbity score  Clashscore  Poor rotamers (%) | 1.36  4.02  0.00 | 1.22  4.39  0.00 | 1.25  4.77  0.00 | 1.17  3.79  0.00 |
| Ramachandran plot  Favored (%)  Allowed (%)  Disallowed (%) | 97.00  3.00  0.00 | 98.21  1.79  0.00 | 98.27  1.73  0.00 | 98.37  1.63  0.00 |

**Supplementary Table 3: Cryo-EM data refinement and validation for the minor conformation (single protomer)**

|  | OFS-Glu  EMD-26986  PDB-8CTD | OFS-Apo_KCl_  EMD-26998  PDB-8CUD | OFS-Apo_NMDG_  EMD-27001  PDB-8CUJ | iOFS-Na^+^  EMD-27007  PDB-8CV3 |
| --- | --- | --- | --- | --- |
| **Data collection and processing** |  |  |  |  |
| Symmetry imposed | C1 | C1 | C1 | C1 |
| Initial particle images (no.) | 1,490,916  (C3-expanded) | 1,214,187  (C3-expanded) | 630,909  (C3-expanded) | 1,559,571  (C3-expanded) |
| Final particle images (no.) | 202,573 protomer | 122,507 protomer | 117,532 protomer | 105,451protomer |
| Map resolution (Å)  FSC threshold | 3.43  0.143 | 2.94  0.143 | 3.04  0.143 | 3.04  0.143 |
| Map resolution range (Å) | 3.01 ~ 32.03 | 2.67 ~ 34.20 | 2.66 ~ 36.59 | 2.69 ~ 41.69 |
|  |  |  |  |  |
| **Refinement** |  |  |  |  |
| Initial model used (PDB code) | 6X2Z | 6X2Z | 6X2Z | 6X2Z |
| Model resolution (Å)  FSC threshold | 3.66  0.5 | 3.15  0.5 | 3.29  0.5 | 3.31  0.5 |
| Model resolution range (Å) | 3.66 ~ 19.55 | 3.15 ~ 18.45 | 3.29 ~ 21.07 | 3.31 ~ 19.27 |
| Map sharpening *B* factor (Å^2^) | -156.8 | -100.7 | -83.5 | -91.8 |
| Model composition  Non-hydrogen atoms  Protein residues  Ligands | 3,130  410  5 | 3,294  433 | 3,294  433 | 3,168  416  2 |
| *B* factors (Å^2^)  Protein  Ligand | 62.17  116.27 | 56.42 | 75.4 | 41.04  36.34 |
| R.m.s. deviations  Bond lengths (Å)  Bond angles (°) | 0.003  0.626 | 0.005  0.667 | 0.002  0.530 | 0.002  0.468 |
| Validation  MolProbity score  Clashscore  Poor rotamers (%) | 1.56  9.77  0.00 | 1.09  2.95  0.00 | 1.35  5.90  0.00 | 1.23  2.76  0.00 |
| Ramachandran plot  Favored (%)  Allowed (%)  Disallowed (%) | 97.77  2.23  0.00 | 98.14  1.86  0.00 | 97.90  2.10  0.00 | 97.07  2.93  0.00 |

**Supplementary Table 4: pKa-s and solvent-accessible surface areas of E374**

| States | OFS-  Apo_KCl_ | OFS-  Apo_NMDG_ | OFS-Na^+^ | OFS-Glu | iOFS-  K^+^ | iOFS- Apo | iOFS-Na^+^ | iOFS^*^-Glu | |
| --- | --- | --- | --- | --- | --- | --- | --- | --- | --- |
| pKa^#^ | 9.2 | 10.0 | 7.6 | 8.9 | 5.5 | 6.5 | 7.1 | 8.4 | |
| ASA (Å^2^)^$^ | 0.2 | 1.6 | 2.1 | 0.0 | 1.3 | 25.3 | 16.2 | 0.0 | |
| ^#^ pKa values calculated using PROPKA for chains A  ^$^ Solvent-accessible surface area (ASA) of E374 calculated using PISA for chains A | | | | | | | | |  |
