# Supplementary material for: Symport and antiport mechanisms of human glutamate transporters
Source: Nat Commun. 2023 May 4;14:2579. doi: 10.1038/s41467-023-38120-5 (PMC10160106; doi:10.1038/s41467-023-38120-5)
Supplement: Supplementary file 3 — Description of Additional Supplementary Files [file 41467_2023_38120_MOESM3_ESM.pdf]

File name: Supplementary Movie 1

Description: Elevator movement of SLC1 transporters. The elevator movements of the transport domain, with HP1 colored blue, HP2 red, and TMs 3, 6, 7, and 8 light green. The scaffold domain TMs 1, 2, 4, and 5 are colored wheat. hEAAT3g protomers in aspartate-bound OFS (PDB ID: 6X2Z [http://doi.org/10.2210/pdb6x2z/pdb]) and Apo IFS (PDB ID: 6X3F [http://doi.org/10.2210/pdb6x3f/pdb]) were used to prepare the movie.

File name: Supplementary Movie 2

Description: Comparison between aspartate- and glutamate-bound hEAAT3 transport domains. Structures of OFS-Asp (PDB ID: 6X2Z [http://doi.org/10.2210/pdb6x2z/pdb]) and iOFS* -Glu are superimposed on the cytoplasmic half of the transport domain (residues 314-372 and 442-465). TMs 3 and 6 are removed for clarity. HP1, TMs 7 and 8 are colored gray, and HP2, substrate, M367 in the NMD motif, and coordinating residues are colored orange and blue, respectively.
