# Supplementary material for: Symport and antiport mechanisms of human glutamate transporters
Source: Nat Commun. 2023 May 4;14:2579. doi: 10.1038/s41467-023-38120-5 (PMC10160106; doi:10.1038/s41467-023-38120-5)
Supplement: Supplementary file 6 — Reporting Summary [file 41467_2023_38120_MOESM6_ESM.pdf]

nature portfolio

Corresponding author(s): Olga Boudker

Last updated by author(s): Mar 21, 2023

Reporting Summary

Nature Portfolio wishes to improve the reproducibility of the work that we publish. This form provides structure for consistency and transparency in reporting. For further information on Nature Portfolio policies, see our Editorial Policies and the Editorial Policy Checklist.

Statistics

For all statistical analyses, confirm that the following items are present in the figure legend, table legend, main text, or Methods section.

n/a	Confirmed	
☐	☒	The exact sample size (*n*) for each experimental group/condition, given as a discrete number and unit of measurement
☐	☒	A statement on whether measurements were taken from distinct samples or whether the same sample was measured repeatedly
☒	☐	The statistical test(s) used AND whether they are one- or two-sided<br>*Only common tests should be described solely by name; describe more complex techniques in the Methods section.*
☒	☐	A description of all covariates tested
☒	☐	A description of any assumptions or corrections, such as tests of normality and adjustment for multiple comparisons
☐	☒	A full description of the statistical parameters including central tendency (e.g. means) or other basic estimates (e.g. regression coefficient) AND variation (e.g. standard deviation) or associated estimates of uncertainty (e.g. confidence intervals)
☒	☐	For null hypothesis testing, the test statistic (e.g. *F*, *t*, *r*) with confidence intervals, effect sizes, degrees of freedom and *P* value noted<br>*Give P values as exact values whenever suitable.*
☒	☐	For Bayesian analysis, information on the choice of priors and Markov chain Monte Carlo settings
☒	☐	For hierarchical and complex designs, identification of the appropriate level for tests and full reporting of outcomes
☒	☐	Estimates of effect sizes (e.g. Cohen's *d*, Pearson's *r*), indicating how they were calculated

Our web collection on statistics for biologists contains articles on many of the points above.

Software and code

Policy information about availability of computer code

Data collection	Legion , Nanion SURFE²R N1
Data analysis	Cryo-EM data sets were processed by MotionCor2, CTFFIND, CryoSPARC v3.2.0, RELION3.1, and pyem v0.5. The models were refined using Coot, and Phenix. Structures were analyzed using ChimeraX-1.2.5, Pymol 2.3.4, CARVER3.0, PROPKA3.0, and PISA v2.1.0. The Solid Supported Membrane transport assay was analyzed using Nanion SURFE²R N1 software.

For manuscripts utilizing custom algorithms or software that are central to the research but not yet described in published literature, software must be made available to editors and reviewers. We strongly encourage code deposition in a community repository (e.g. GitHub). See the Nature Portfolio guidelines for submitting code & software for further information.

Data

Policy information about availability of data

All manuscripts must include a data availability statement. This statement should provide the following information, where applicable:

- Accession codes, unique identifiers, or web links for publicly available datasets
- A description of any restrictions on data availability
- For clinical datasets or third party data, please ensure that the statement adheres to our policy

The cryo-EM maps and atomic coordinates generated in this study have been deposited in the Electron Microscopy Data Bank (EMDB) and Protein Data Bank (PDB) under accession codes EMD-26985 (iOFS*-Glu, PDB-8CTC), EMD-26986 (OFS-Glu, PDB-8CTD), EMD-26997 (iOFS-K+, PDB-8CUA), EMD-26998 (OFS-ApoKCl,

PDB-8CUD), EMD-27000 (iOFS-Apo, PDB-8CUI), EMD-27001 (OFS-ApoNMDG, PDB-8CUJ), EMD-27006 (OFS-Na+ bound, PDB-8CV2), and EMD-27007 (iOFS-Na+ bound, PDB-8CV3). The models and cryo-EM maps (PDB ID: 4P1A, 6UWL, 6X2Z, 6X2L, 6X3F, EMD-22022, EMD-22023, EMD-22024) used in the research are adapted from PDB and EMDB respectively. Source data is provided with this paper.

Human research participants

Policy information about studies involving human research participants and Sex and Gender in Research.

Reporting on sex and gender	n/a
Population characteristics	n/a
Recruitment	n/a
Ethics oversight	n/a

Note that full information on the approval of the study protocol must also be provided in the manuscript.

Field-specific reporting

Please select the one below that is the best fit for your research. If you are not sure, read the appropriate sections before making your selection.

☒ Life sciences ☐ Behavioural & social sciences ☐ Ecological, evolutionary & environmental sciences

For a reference copy of the document with all sections, see nature.com/documents/nr-reporting-summary-flat.pdf

Life sciences study design

All studies must disclose on these points even when the disclosure is negative.

Sample size	The cryo-EM data size (number of the movies) in each data set is enough for structure determination in this project based on our experience.
Data exclusions	No data were excluded from analyses.
Replication	For the Solid Supported Membrane (SSM) assay, at least two sensors for each proteoliposome preparation were recorded, producing similar results. The cellular uptake assays were performed 3 times for each construct using independently prepared samples, each with a technical duplicate. All attempts at replication were successful.
Randomization	Randomization was used in cryo-EM refinement (RELION3) and it was random. The sensors used for SSM assay were randomly selected, the currents measured for different sensors are not quantitatively comparable because of the principle of the assay.
Blinding	Blinding is not relevant to cryo-EM, SSM, cellular uptake assay.

Reporting for specific materials, systems and methods

We require information from authors about some types of materials, experimental systems and methods used in many studies. Here, indicate whether each material, system or method listed is relevant to your study. If you are not sure if a list item applies to your research, read the appropriate section before selecting a response.

Materials & experimental systems

n/a	Involved in the study	
☒	☐	Antibodies
☐	☒	Eukaryotic cell lines
☒	☐	Palaeontology and archaeology
☒	☐	Animals and other organisms
☒	☐	Clinical data
☒	☐	Dual use research of concern

Methods

n/a	Involved in the study	
☒	☐	ChIP-seq
☒	☐	Flow cytometry
☒	☐	MRI-based neuroimaging

Eukaryotic cell lines

Policy information about cell lines and Sex and Gender in Research

Cell line source(s)	FreeStyle 293F cells (Invitrogen R79007) was purchased from Thermo Fisher Scientific.
Authentication	The FreeStyle™ 293-F cell line was purchased from vendor. Cell line Authentication was not performed.
Mycoplasma contamination	Cell lines were not tested for mycoplasma contamination.
Commonly misidentified lines (See ICLAC register)	n/a
